# Paternal Genetic Contribution Influences Fetal Vulnerability to Maternal Alcohol Consumption in a Rat Model of Fetal Alcohol Spectrum Disorder

**DOI:** 10.1371/journal.pone.0010058

**Published:** 2010-04-07

**Authors:** Laura J. Sittig, Eva E. Redei

**Affiliations:** Asher Center, Department of Psychiatry and Behavioral Sciences, Feinberg School of Medicine, Northwestern University, Chicago, Illinois, United States of America; City of Hope National Medical Center, United States of America

## Abstract

**Background:**

Fetal alcohol exposure causes in the offspring a collection of permanent physiological and neuropsychological deficits collectively termed Fetal Alcohol Spectrum Disorder (FASD). The timing and amount of exposure cannot fully explain the substantial variability among affected individuals, pointing to genetic influences that mediate fetal vulnerability. However, the aspects of vulnerability that depend on the mother, the father, or both, are not known.

**Methodology/Principal Findings:**

Using the outbred Sprague-Dawley (SD) and inbred Brown Norway (BN) rat strains as well as their reciprocal crosses, we administered ethanol (E), pair-fed (PF), or control (C) diets to the pregnant dams. The dams' plasma levels of free thyroxine (fT4), triiodothyronine (T3), free T3 (fT3), and thyroid stimulating hormone (TSH) were measured to elucidate potential differences in maternal thyroid hormonal environment, which affects specific aspects of FASD. We then compared alcohol-exposed, pair fed, and control offspring of each fetal strain on gestational day 21 (G21) to identify maternal and paternal genetic effects on bodyweight and placental weight of male and female fetuses.

**Conclusions:**

SD and BN dams exhibited different baseline hypothalamic-pituitary-thyroid function. Moreover, the thyroid function of SD dams was more severely affected by alcohol consumption while that of BN dams was relatively resistant. This novel finding suggests that genetic differences in maternal thyroid function are one source of maternal genetic effects on fetal vulnerability to FASD. The fetal vulnerability to decreased bodyweight after alcohol exposure depended on the genetic contribution of both parents, not only maternal contribution as previously thought. In contrast, the effect of maternal alcohol consumption on placental weight was consistent and not strain-dependent. Interestingly, placental weight in fetuses with different paternal genetic contributions exhibited opposite responses to caloric restriction (pair feeding). In summary, these novel findings demonstrate both maternal and paternal genetic contributions to *in utero* vulnerability to alcohol, refining our understanding of the genetically-based heterogeneity seen in human FASD.

## Introduction

Alcohol consumption during pregnancy can result in fetal alcohol spectrum disorder (FASD), a constellation of disabilities including deficient pre- and postnatal growth, morphological malformations of the face and/or brain, and cognitive and behavioral deficits [1]. These teratological outcomes vary significantly among individuals with respect to range and severity even after allowing for the effects of timing, duration, and amount of alcohol exposure, suggesting that genetic vulnerability may contribute to the etiology of FASD [2]. Human studies have implicated a role for maternal genotype for alcohol dehydrogenase 1B and some have agreed that the ADH1B*3 allele confers some protection to the fetus [3], [4] but these or other maternal genetic factors are not the sole source of genetic vulnerability since dizygotic twins can be discordant for FAS diagnosis [5]–[7]. Animal studies using a single dosage of ethanol during pregnancy show that different mouse strains exhibit different severities of morphological and fetal weight deficits after alcohol exposure, which the authors attributed primarily to maternal genetic effects [8]–[10]. However, these data together with the observations in human alcohol-exposed twins also suggest that paternal genetic influences may directly impact fetal susceptibility to alcohol. We therefore hypothesized that maternal and paternal genetic factors interact to determine the extent of teratogenesis to the fetus after chronic prenatal alcohol exposure.

The rat is commonly used as a model for prenatal alcohol exposure because pregnant females voluntarily consume a diet containing a moderate amount of alcohol, resulting in physiological and behavioral deficits in the offspring that parallel those seen in human FASD. Previously we have characterized the fetal and maternal outcome of this paradigm using the Sprague-Dawley (SD) rat strain. Alcohol-consuming SD dams exhibit suppressed thyroid hormone status and their fetuses have decreased bodyweight, increased placental weight, and learning and memory deficits which are partially due to alcohol-induced maternal thyroid deficiency during gestation [11]–[13]. Thyroid hormone economy contributes to programming of fetal behavior and hormonal function, and normalization of maternal thyroid status reverses some neurodevelopmental consequences of prenatal alcohol exposure in the adult offspring [12]. Thus, maternal genetic influences controlling thyroid hormone economy are likely to contribute to genetic vulnerability to alcohol, or by the same token could alleviate adverse effects by conferring resilience.

Here we explored maternal genetic influences and potential maternal-paternal genetic interactions that could modulate adverse effects of maternal alcohol consumption on the fetus. To this end we employed pure strains and reciprocal crosses of two genetically and physiologically distinct rat strains, and administered ethanol, pair-fed, and control diets to the pregnant dams. We then analyzed maternal thyroid status as well as fetal and placental weight changes in response to prenatal alcohol exposure. We chose to use the Brown Norway (BN), the most phylogenetically divergent inbred rat strain, and the Sprague Dawley (SD), the most commonly utilized outbred strain [14]. The BN and SD genomes have been sequenced by the Rat Genome Project and Celera respectively, and differences between these strains in litter size, temperament, appearance, and attributes of sensorimotor gating have been reported [14].

## Methods

### Animals

All animal experimentation was carried out in accordance with the NIH guide for the care and use of laboratory animals and approved by the Northwestern University Animal Care and Use Committee. Adult Sprague-Dawley (SD) and Brown-Norway (BN) males and females (Harlan, Indianapolis, IN) were housed by strain, in groups (females) or individually (males) in a temperature- and humidity-controlled vivarium with regular light/dark cycles (on at 0600 h, off at 1800 h). After one week of acclimatization, SD and BN females were mated with an SD or BN male overnight. Gestational day 1 (G1) was designated upon the presence of sperm in vaginal smears and pregnant females were assigned to a diet group on G8: E (ethanol), PF (pair-fed), or C (control). Average maternal age and weight at pregnancy was SD: 12–14 weeks, 215.0±1.4 g; BN: 17–20 weeks, 151.6±3.4 g.

### E, PF, and C Diets

Maternal diet procedures were performed as described previously [13]. E dams (n = 5–6/cross) received an ethanol-containing (5% w/v, 35% ethanol-derived calories) liquid diet (Lieber-DeCarli'82, BioServ) for the last two weeks of gestation as described previously [15]. Pair-fed dams (n = 5–8/cross) received an amount of isocaloric liquid diet (Lieber DeCarli'82, BioServ) that matched the paired E dam's diet consumption on the previous day. SD and BN females ate the same amount of ethanol diet per bodyweight from G11–G21 (data not shown) and there was no difference in blood alcohol levels between SD and BN dams (SD: 126.5±29.0 mg/dl; BN: 125.8±17.5 mg/dl). The PF dams are food-restricted compared to controls, but are the most well-accepted control group for decreased calorie intake of E dams [16]. Control dams (n = 5–8/cross) received lab chow and water *ad libitum*. Pregnant dams were killed by decapitation on G21 between 1000 h and 1200 h, and the uterine horn was quickly removed onto ice. Fetuses were removed individually, sexed according to anogenital distance, and their bodyweight and placental weight were recorded. Depending on litter sizes, means for fetal weight and placental weight were based on n = 3–4 litters for SD/SD and SD/BN crosses, and n = 4–6 litters for BN/SD and BN/BN crosses. Maternal blood was collected on ice into EDTA-containing tubes and centrifuged to obtain plasma that was stored at −80 C until use.

### Radioimmunoassays

Single assays for maternal TSH, T3, free T3, and free T4 were conducted using plasma collected on G21. A rat TSH RIA manufactured by Alpco Diagnostics (Salem, NH) was used where assay sensitivity was 1.6 ng/ml and intra-assay CV 3.9%. T3, fT3, and fT4 assays were manufactured by MP Biomedicals, LLC (Irvine, CA) and assay sensitivity/CVs were (T3: 36 ng/dl, 9.5%; fT3: 0.6 pg/ml, 8%; fT4: 0.2 ng/dl, 3.8%).

## Results

### Maternal Measures

**Figure 1:**
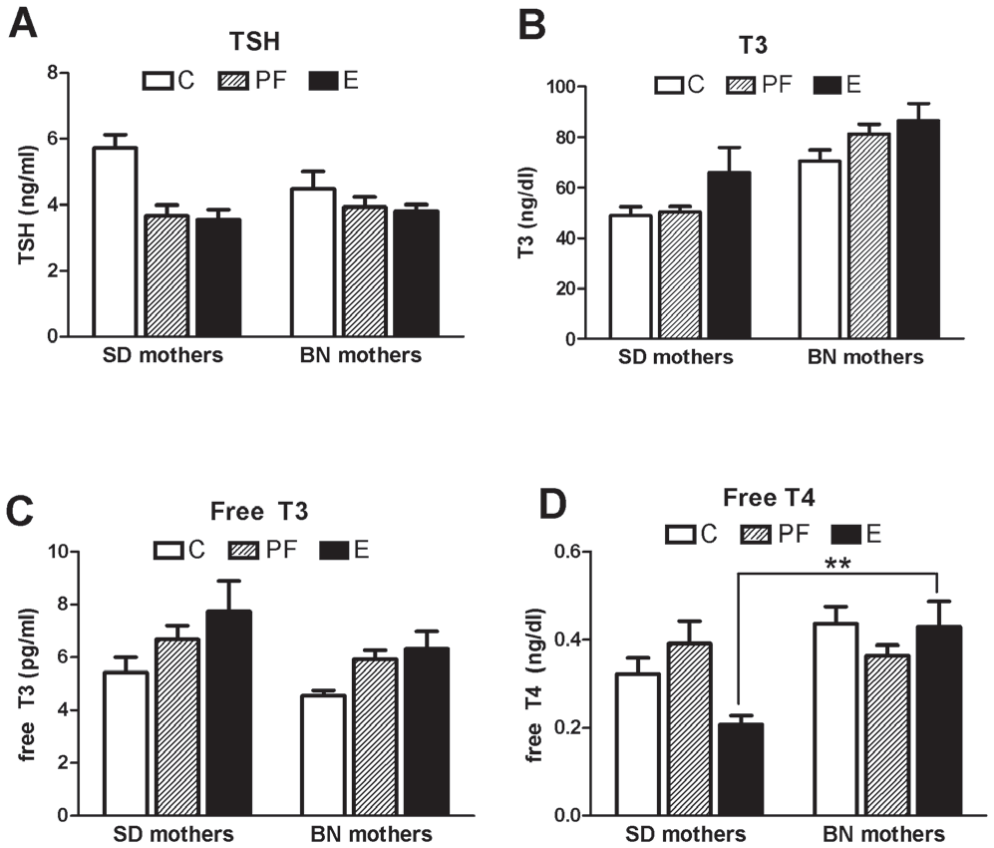
Pregnant SD and BN dams exhibit different susceptibility to alcohol in their thyroid function. (A) Plasma TSH, (B) total T3, (C) free T3 and (D) free T4 levels in control (C), pair-fed (PF), and ethanol-consuming (E) mothers on gestational day 21 (G21). SD mothers include those mated to SD or BN males; BN mothers include those mated to SD or BN males. Alcohol-containing and calorie-restricted diets suppressed plasma TSH levels (F [2, 57] = 9.3, *P*<0.01) and increased T3 (F [2, 58] = 4.0, *P* = 0.02) and fT3 (F [2, 49] = 5.6, *P*<0.01) levels. Plasma fT4 was decreased by alcohol consumption in SD but not BN mothers (F [2, 56] = 5.5, *P*<0.01). Plasma T3 levels (F [1, 58] = 28.0, *P*<0.001) and fT4 (F [1, 56] = 11.1, *P*<0.01) were higher in BN mothers than SD mothers while fT3 levels were lower in BN (F [1, 49] = 4.1, *P*<0.05). All data (means {plus minus} SEM) were analyzed by two-way ANOVAs and Bonferroni post-hoc results are shown (**P<0.01).

Based on our previous finding that maternal thyroid status is causative in alcohol-related deficits, we measured thyroid hormones of E, PF and C SD and BN dams on G21. Maternal plasma fT4, fT3, T3 and TSH measures were pooled by maternal strain and analyzed by two-way ANOVAs (Figure 1). BN females had higher plasma fT4 levels (maternal strain: F [1, 56] = 11.1, *P*<0.01) and higher plasma T3 levels (maternal strain: F [1, 58] = 28.0, *P<0*.001) than SD females. On the other hand, BN dams had lower fT3 levels than SD (maternal strain: F [1, 49] = 4.1, *P*<0.05) but no difference in plasma TSH levels (maternal strain: F [1, 57] = 0.70, *P* = 0.42). Ethanol consumption suppressed plasma fT4 levels in SD females, while BN females were fully resistant to this effect of alcohol (maternal strain x diet: F [2, 56] = 5.5, *P*<0.01). Despite this strain difference in response to ethanol consumption, E and PF diet consumption increased plasma T3 levels in both strains (diet: F [2, 58] = 4.0, *P* = 0.02). Plasma fT3 levels were also elevated in diet-consuming females (diet: F [2, 49] = 5.6, *P*<0.01). As expected, SD females tended to respond to PF and E diets with greater reduction in plasma TSH levels (diet: F [2, 57] = 9.3, *P*<0.01) although the greater vulnerability of SD mothers did not reach significance (maternal strain x diet: F [2, 57] = 3.8, *P* = 0.07). The increased plasma T3 and fT3 in the alcohol-consuming SD has not been found previously [11] but may be attributable to significant weight differences of the females used for these two studies.

#### Litters

By reciprocally crossing SD and BN rats we obtained four fetal strains: SD/SD, SD/BN, BN/SD and BN/BN where maternal strain is first. Respective litter sizes and sex ratios are given by fetal strain in Table 1. Similar to a previous report, SD dams had larger litters than BN dams (maternal strain: F [1, 55] = 155.1, *P*<0.001) [14]. There was no effect of maternal diet on litter size (F [2, 55] = 0.19, *P*>0.05). The number of male fetuses was decreased in SD mothers consuming E or PF diets (diet: F [2], [28] = 4.1, *P* = 0.03). The paternal genetic contribution did not affect the number of male and female fetuses per litter for either SD or BN mothers (not shown).

**Table 1:** Litter sizes and male/female ratios obtained from SD and BN litters and hybrid crosses.

Fetal	Genotype	Control	Pair fed	Ethanol
**SS**	***litter size	14.2±0.7	11.0±2.3	12.8±0.9
	males/females	7.8±1.0**/**6.3±0.3	5.0±0.9/5.7±1.5	6.0±0.9/6.7±1.7
**SB**	***litter size	15.0±0.7	12.2±1.8	12.8±1.6
	males/females	7.7±0.8/7.3±0.9	5.2±1.4/7.0±1.4	5.8±1.1/7.0±0.9
**BS**	litter size	4.0±0.6	5.1±0.9	3.3±0.6
	males/females	1.8±0.4/2.2±0.7	3.1±0.6/2.0±0.5	1.8±0.4/1.5±0.6
**BB**	litter size	5.5±0.3	4.3±0.9	4.7±0.6
	males/females	3.2±0.5/2.2±0.8	2.8±0.5/1.5±0.4	3.2±1.0/1.5±0.4

***SD dams' litter sizes are larger than BN, *P*<0.001.

### Fetal measures

#### Fetal weight

**Figure 2:**
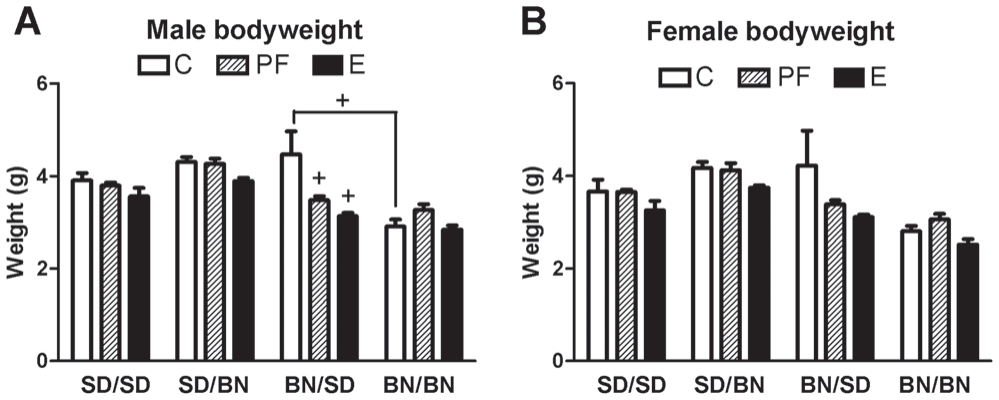
Diet effects on fetal bodyweight are modulated by maternal and paternal genetic backgrounds. Fetal bodyweight of C, PF, and E male and female fetuses (SD/SD, SD/BN, BN/SD, and BN/BN where maternal strain is first) on G21. Means from individual litters were used to calculate group final means ± SEM as shown. Males (A) and females (B) were separated due to the effect of fetal sex on bodyweight (F [1, 71] = 4.5, *P* = 0.04). Fetuses obtained from hybrid crosses weighed more than pure-strain fetuses on G21 (F [1, 71] = 53.0, *P*<0.001). Alcohol exposure decreased fetal weight (F [2, 71] = 15.7, *P*<0.001), but BN/BN fetuses were not as vulnerable as BN/SD fetuses (maternal strain x paternal strain x diet: F [2, 71] = 5.6, *P*<0.01). Data were analyzed by four-way ANOVA with sex as a factor. Planned comparisons were made with Bonferroni adjustment (+*P*<0.0001). Comparisons are relative to the control group of the same fetal strain unless otherwise indicated.

Male fetuses weighed more than females (sex: F [1, 71] = 4.5, *P* = 0.04) so their weights are presented individually (Figure 2). Fetuses of SD mothers were heavier than those carried by BN mothers (maternal strain: F [1, 71] = 55.4, *P*<0.001). The offspring of SD mothers weighed more when the father was BN, while offspring of BN mothers weighed less when the father was BN (maternal strain x paternal strain: F [1, 71] = 53.0, *P*<0.001). The response to pair-feeding also depended on parental strains: PF decreased the bodyweight of BN/SD fetuses but not SD/SD or SD/BN fetuses. In contrast, pure BN fetuses showed increased bodyweight after pair-feeding (maternal strain x paternal strain x diet: F [2, 71] = 5.6, *P*<0.01).

**Figure 3:**
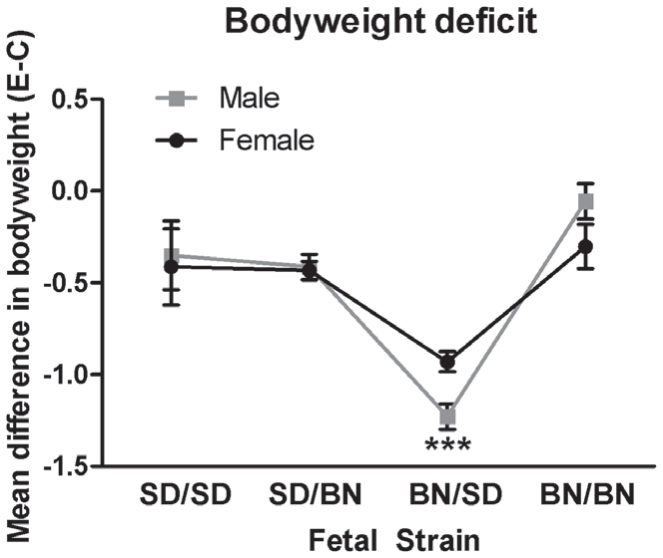
Unique bodyweight deficit of alcohol-exposed BN/SD illustrates the interaction of both parental genetic backgrounds in fetal vulnerability to alcohol. The difference in mean bodyweight between control and alcohol-exposed male and female fetuses is shown for the pure and hybrid fetal strains. There was no significant effect of fetal sex on bodyweight deficits; therefore, data were analyzed by one-way ANOVA with fetal strain as the factor. Bodyweight deficits exhibited by BN/SD fetuses were significantly greater than all other strains (fetal strain: F [3], [30] = 25.8, *P*<0.0001) including the BN/BN fetuses of the same mother, illustrating a maternal-paternal interaction of genetic background. *** *P*<0.001 by Bonferroni post-hoc test.

Overall, alcohol exposure decreased fetal weight below that of PF and controls (diet: F [2, 71] = 15.7, *P*<0.001). BN/BN fetuses were relatively resistant to the effect of alcohol on bodyweight, consistent with the BN dams' resistance to thyroid hormone alterations (Figure 1). In contrast, BN/SD fetal bodyweight was more severely affected by alcohol exposure compared to the other fetal strains, an SD paternal effect only apparent when the mother was BN (maternal strain x paternal strain x diet: F [2, 71] = 5.6, *P*<0.01). Alcohol-induced decreases in fetal bodyweight compared to the respective controls are summarized for clarity in Figure 3, showing that the BN/SD deficit in bodyweight was exaggerated compared to the others (fetal strain: F [3], [30] = 25.8, *P*<0.0001).

#### Placental weight

**Figure 4:**
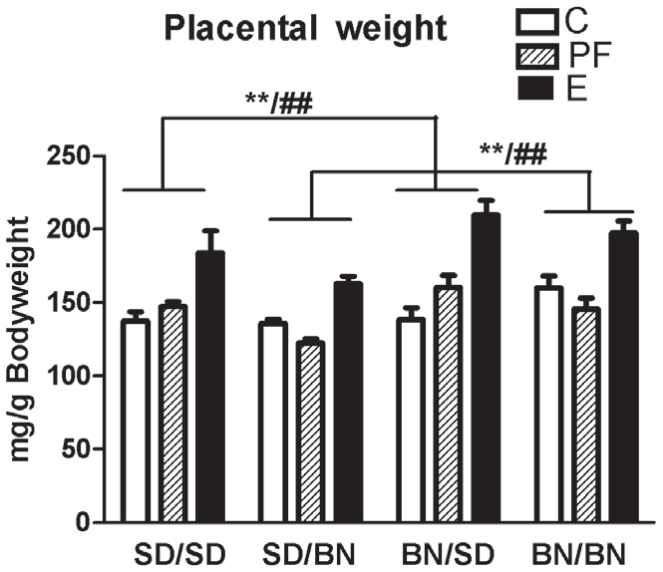
Pair-feeding effect on placental weight depends on strain while alcohol effect does not. Placental weight of C, PF, and E fetuses (SD/SD, SD/BN, BN/SD, and BN/BN where maternal strain is first) on G21. Placental weights were normalized to fetal bodyweights and means from individual litters were used to calculate group means ± SEM as shown. There was no effect of fetal sex, so male and female data were pooled and analyzed by three-way ANOVA. Placentae of BN mothers were heavier than SD (F [1, 85] = 17.5, *P*<0.001). Pair fed placentae of SD fathers were heavier than controls, while pair fed placentae of BN fathers weighed less (paternal strain x diet F [2, 85] = 3.8, *P* = 0.03). In contrast, alcohol exposure increased placental weight regardless of strain (F [2, 85] = 38.0, *P*<0.001). Result of Bonferroni post-hoc test is shown: **E vs. C with SD or BN father regardless of maternal strain (*P*<0.01). ##E vs. PF with SD or BN father regardless of maternal strain (*P*<0.01).

By convention placental weight was normalized to bodyweight of the fetus and we refer to these values as the “placental weight”. There was no effect of fetal sex on placental weight (fetal sex: F [1, 97] = 1.7, *P* = 0.20), so the male and female values were pooled and analyzed by a three-way ANOVA (Figure 4). Placentae of BN mothers were heavier than those of SD mothers (maternal strain: F [1, 85] = 17.5, *P*<0.001), and there was a trend whereby paternal genetic contribution had the opposite effect on placental weight (paternal strain: F [1, 85] = 3.3, *P* = 0.07). Pair feeding had very different effects on placental weight depending on paternal strain, increasing it when the father was SD, but decreasing it when the father was BN (paternal strain x diet: F [2, 85] = 3.8, *P* = 0.03). In contrast, alcohol exposure consistently increased placental weights independent of fetal strain (diet: F [2, 85] = 38.0, *P*<0.001).

## Discussion

Here we present novel findings of maternal and maternal-paternal interactive genetic contributions to fetal vulnerability to alcohol exposure. Maternal effects manifested in a decreased sensitivity of BN dams to disruptions of thyroid function by alcohol consumption compared to SD dams. Furthermore, we show a clear maternal-paternal genetic interaction in determining the fetal vulnerability to alcohol-induced bodyweight deficit whereby one hybrid cross only showed a unique vulnerability, indicating that maternal and paternal strain effects alone cannot explain this fetal response to alcohol.

Differences in thyroid hormone economy could be a component of maternal genetic effects in alcohol teratogenesis. In general, BN dams had higher levels of plasma fT4 and T3 and lower levels of fT3 than SD, which may be due to differing genetic regulation of plasma TSH and/or sensitivity to thyroid hormones [17], [18]. Moreover, SD and BN dams responded differently to alcohol consumption. Specifically, alcohol consumption suppressed fT4 only in the SD dams while BN were completely unaffected. This was not due to diet consumption differences as neither blood alcohol levels nor alcohol diet consumption differed between strains, suggesting a genetic cause. If accordingly less maternal fT4 is transported through the placenta to the fetus [19], alcohol-exposed fetuses of SD mothers experience a more adversely affected thyroid hormone milieu compared to those of BN mothers. The positive association of maternal fT4 levels with birth weight [20], [21] suggests that the BN dam's unaffected fT4 levels are related to the normal bodyweight of alcohol-exposed male BN/BN fetuses.

The fact that SD dams had larger litters than BN dams supports the finding that maternal strain rather than fetal strain is the major determinant of litter size [22], but genetic effects on fetal bodyweight were more complex. Apart from the maternal genetic effect that SD-mothered fetuses were heavier than BN, hybrid cross SD/BN and BN/SD control fetuses were heavier than those of either homozygous cross, indicating some degree of overdominance for the trait. The same hybrid weight advantage was noted in hybrid mouse fetuses but was not attributed to overdominance by the authors [8], [9]. Several types of genetic influence contribute to bodyweight such that additive and dominant modes of inheritance principally control bodyweight from birth to post-weaning age [23]. Additive genetic effects explain approximately 75% of birth weight variation during the first postnatal week while dominant genetic patterns including overdominance account for approximately 25% [23]. Alternatively, a role for genetic imprinting in the observed pattern of fetal bodyweight cannot be ruled out as at least one paternally-expressed locus has been linked to inheritance of birth weight in a human population, and a candidate imprinted locus contributing to bodyweight has been identified on mouse chromosome 14 [24], [25].

Previous studies have attributed fetal susceptibility to alcohol-induced weight deficit to maternal genotype effects only [8], [9]. In contrast, we found that maternal and paternal genetic contributions interact to determine the fetal response to alcohol exposure. BN/SD fetuses had a larger alcohol-induced bodyweight deficit compared to the genetically similar SD/BN fetuses or the pure-strain BN/BN fetuses. This differential pattern of alcohol vulnerability in the reciprocal hybrids emulates the classic phenotypic “signature” of imprinted parent-of-origin effects. Thus, the heightened vulnerability of the BN/SD strain to fetal weight deficit could result from alcohol-induced alterations of epigenetic marks at imprinted loci. If so, this would indicate that the fetus remains epigenetically sensitive to alcohol beyond the early stages marked by preconception, implantation and gastrulation, as previously shown [26], [27]. While imprinting effects can often be confounded by maternal genetic effects on the intrauterine environment [28], [29], the alcohol-exposed BN/BN fetuses of this study shared the same intrauterine environment as the most vulnerable BN/SD but unlike BN/SD, exhibited the mildest bodyweight deficit. The possibility that imprinted loci contributing to bodyweight may influence the long-term effects of alcohol could be tested using novel candidate imprinted loci regulating bodyweight [24], [25].

We found that near-term placental weight increased after maternal alcohol consumption regardless of maternal or paternal genetic background. Placental function is critical for controlling fetal growth, and a higher placental weight relative to birth weight is associated with future cardiovascular mortality and metabolic dysfunction in adulthood [30], [31]. While the stability of increased placental weight as a potential end-of-term “marker” for prenatal alcohol exposure should be verified in further rodent strains and humans, the finding is consistent with our past study [13], and placental dysfunction occurs in alcohol-consuming humans and rodents [32]–[34]. Such characteristics of alcohol-exposed placental tissue could support the identification of biomarkers for prenatal alcohol exposure, an important prospect for clinicians who may not obtain accurate information due to the social stigma preventing women from accurately reporting their alcohol use and a lack of diagnostic laboratory tests [35]. Apart from the alcohol effects on the placenta, we also found that placental weights of the control and pair-fed fetuses of the hybrid fetal strains reflected opposing maternal and paternal influences. Maternal strain significantly influenced placental weight while the paternal influence tended to affect it in the opposite direction; moreover, the paternal strain determined whether pair-feeding of the mother increased or decreased placental weight. These findings may relate to the role of imprinted genes in the growth of the placenta whereby paternally expressed genes enhance and maternally expressed genes restrict placental growth [36].

We have demonstrated an interactive maternal-paternal genetic influence on the fetal vulnerability to alcohol-induced decrease in bodyweight. Future studies should address the extent to which bodyweight and placental weight predict long-term deficits independently from the ability of the postnatal social environment to accentuate or ameliorate alcohol-induced disability [37]. Further, fruitful investigations could target the specific genetic loci that underlie the parent-of-origin dependency of alcohol effects. In a larger context, our findings emphasize the potential malleability of seemingly canonical environmental effects by genetics. Further inquiries into how prenatal environmental insults such as alcohol exposure interact with genetic background will inform the analysis of many complex human diseases.
